# Therapeutic equine hyperimmune antibodies with high and broad-spectrum neutralizing activity protect rodents against SARS-CoV-2 infection

**DOI:** 10.3389/fimmu.2023.1066730

**Published:** 2023-02-17

**Authors:** Entao Li, Qiuxue Han, Jinhao Bi, Shimeng Wei, Shen Wang, Ying Zhang, Jun Liu, Na Feng, Tiecheng Wang, Jun Wu, Songtao Yang, Yongkun Zhao, Bo Liu, Feihu Yan, Xianzhu Xia

**Affiliations:** ^1^ Changchun Veterinary Research Institute, Chinese Academy of Agricultural Sciences, Changchun, China; ^2^ College of Veterinary Medicine, Jilin Agriculture University, Changchun, China; ^3^ Institute of Laboratory Animal Science, Chinese Academy of Medical Science and Comparative Medicine Center, Peking Union Medical College, Beijing, China; ^4^ College of Wildlife and Protected Area, Northeast Forestry University, Harbin, Heilongjiang, China; ^5^ Department of Microorganism Engineering, Beijing Institute of Biotechnology, Beijing, China

**Keywords:** SARS-CoV-2, equine antibody, variants of concern, variants of interest, broad-spectrum neutralizing activity

## Abstract

The emergence of SARS-CoV-2 variants stresses the continued need for broad-spectrum therapeutic antibodies. Several therapeutic monoclonal antibodies or cocktails have been introduced for clinical use. However, unremitting emerging SARS-CoV-2 variants showed reduced neutralizing efficacy by vaccine induced polyclonal antibodies or therapeutic monoclonal antibodies. In our study, polyclonal antibodies and F(ab’)_2_ fragments with strong affinity produced after equine immunization with RBD proteins produced strong affinity. Notably, specific equine IgG and F(ab’)_2_ have broad and high neutralizing activity against parental virus, all SARS-CoV-2 variants of concern (VOCs), including B.1.1,7, B.1.351, B.1.617.2, P.1, B.1.1.529 and BA.2, and all variants of interest (VOIs) including B.1.429, P.2, B.1.525, P.3, B.1.526, B.1.617.1, C.37 and B.1.621. Although some variants weaken the neutralizing ability of equine IgG and F(ab’)_2_ fragments, they still exhibited superior neutralization ability against mutants compared to some reported monoclonal antibodies. Furthermore, we tested the pre-exposure and post-exposure protective efficacy of the equine immunoglobulin IgG and F(ab’)_2_ fragments in lethal mouse and susceptible golden hamster models. Equine immunoglobulin IgG and F(ab’)_2_ fragments effectively neutralized SARS-CoV-2 *in vitro*, fully protected BALB/c mice from the lethal challenge, and reduced golden hamster’s lung pathological change. Therefore, equine pAbs are an adequate, broad coverage, affordable and scalable potential clinical immunotherapy for COVID-19, particularly for SARS-CoV-2 VOCs or VOIs.

## Introduction

Severe acute respiratory syndrome coronavirus 2 (SARS-CoV-2) is a causative agent of coronavirus disease 2019 (COVID-19) discovered in December 2019 (1). SARS-CoV-2 is a single-stranded enveloped, positive-sense RNA virus. The virion is mainly composed of membrane protein (M), and nucleocapsid protein (N), spike protein (S), and envelope protein (E) (2). The S protein is a highly glycosylated type I membrane protein composed of S1 and S2 subunits on the surface of the virus particle. It is displayed on the surface of the virus particle in the form of a homotrimer, forming a unique coronavirus S structure that mediates virus entry (3). The receptor binding domain (RBD) is located on the S1 subunit, which mediates SARS-CoV-2 binding to the angiotensin-converting enzyme 2 (ACE2) receptor of the host cell (4) and acts as an antigenic epitope inducing neutralizing antibodies (5, 6). The function of the transmembrane unit S2 is to promote membrane fusion between the virus and the host cell (7, 8). The above characteristics of the S protein make it an important target for the development of vaccines and antibodies against SARS-CoV-2 (9). In the course of the COVID-19 pandemic, the host spectrum of the virus has been expanding and the cellular microenvironment has been different. This can lead the virus to make a series of adaptive mutations to adapt to their hosts, which influence its virulence, transmissibility, or pathogenesis (10). Numerous SARS-CoV-2 variants have already been documented globally during the COVID-19 pandemic, classified as VOC and VOI, and high consequence (11). Five variants have rapidly become dominant in several countries, raising concerns as they possess mutations of interest and providing evidence of international spreading: B.1.1,7 (Alpha), B.1.351 (Beta), B.1.617.2 (Delta), P.1 (Gamma), and B.1.1.529 (Omicron) (12). The strike of the increasing number of confirmed cases and deaths globally is unimaginable. Although a variety of therapeutic antibody drugs, such as homologous or heterologous monoclonal antibodies (13–15) or their cocktails (16), nanobodies (17–20), humanized antibodies (21) and chimeric antibodies (22), have been licensed or approved to enter clinical trials, the current epidemic situation and the emergence of SARS-CoV-2 variants have prompted the acceleration of the development of therapeutic antibody drugs with broad-spectrum neutralizing activity (23, 24).

At the early stage of the COVID-19 global pandemic, plasma transfusion from recovered individuals was identified as a potential therapeutic method for COVID-19 in clinical practice (25–29). Obviously, the quantity and quality of plasma were not guaranteed to satisfy the standard of clinical use, limiting the widespread use of this treatment method (30–32). Immunoglobulin purified from COVID-19 convalescent plasma showed higher activity than the original plasma (33). However, treatments relying on human plasma have obvious and neglected shortcomings. Blood sources are highly dependent on human volunteers, and human pathogens have to be screened. It is also worth noting that the nucleic acid of SARS-CoV-2 can be detected in the plasma, and the risk of transmission through blood transfusion still exists. Fortunately, animal-derived immunoglobulins can solve the problem of a lack of blood sources. Antiserum produced after active immunization of large animals such as horses has long been approved for clinical application to treat complex and difficult diseases, especially venomous snake bites and various highly pathogenic infectious diseases (34–37).

With no licensed antibodies with broad-spectrum neutralizing activity against various SARS-CoV-2 variants, there is an urgent, cross-reactive and affordable therapeutic approach to improve the prognosis of COVID-19 patients and reduce person-to-person spread. Aninsworth S et al. proposed that animal-derived antibodies should be considered alongside convalescent human plasma to deliver treatments for COVID-19 (38). Therefore, another promising therapy for COVID-19 patients is the intravenous administration of heterologous pAbs, which are purified from the plasma of horse hyperimmunized with SARS-CoV-2-related antigens. In this study, we described an application of equine anti-SARS-CoV-2 purified polyclonal IgG and F(ab’)_2_ with demonstration of their cross-reactive neutralizing activity against all SARS-CoV-2 VOCs and VOIs and evaluated their protective efficacy in a BALB/c and hamster model of COVID-19, respectively.

## Results

### Generation of the recombinant RBD protein of SARS-CoV-2

To effectively induce a robust nAb response against SARS-CoV-2 *in vivo*, the RBD of the S protein was selected as the immunogen (**Supplementary Data**, **Figure S1A**). Based on our previous study (39), honeybee melittin (HBM) signal peptide was added to the N-terminal of the RBD region to promote the expression of SARS-CoV-2 RBD protein in secretory forms. Baculovirus expression system and 6×His tags were applied for mass expression and purification of the recombinant RBD protein. As shown in **Supplemental Figure 1**, Western blot results suggested that the recombinant RBD protein was represented by a 35 kDa band (**Figure S1C**). Meanwhile, the purity of the recombinant RBD preparation, as determined by SDS‒PAGE followed by thin layer chromatography, was determined to be 90% (**Figure S1D**). The above results show that the RBD protein of SARS-CoV-2 was well prepared for further research.

### Equine immunization and production of equine antisera products

Three horses (No. 15, No. 16, and No. 17) were immunized with adjuvanted RBD protein purified from Sf9 cells. The results showed that the neutralizing antibody titers of the sera from the three horses all showed a trend of an unremitting increase in the number following a series of immunizations. The neutralizing serum titer was detected with the SARS-CoV-2 Wuhan01 strain in the BSL-3 laboratory. The neutralizing antibody titers of the three horses were 1:34,000, 1:51,200, and 1:25,600 after five immunizations, respectively (**Figure S2B**). In view of the above results, the sera of horses No. 15 and No. 16 after five immunizations were selected for subsequent IgG and F(ab’)_2_ purification. The saturated ammonium sulfate precipitation method was applied to purify IgG from equine serum, and pepsin was used to digest the Fc region to obtain purified F(ab’)_2_ fragments. Finally, the sample was run through a Protein A column to remove the residual immunoglobulin G, and the purified F(ab’)_2_ fragments were collected by running through a Protein-G column. The integrity of IgG and F(ab’)_2_ fragments was determined by SDS‒PAGE (**Figures S2C, D**). After purification, the purities of IgG and F(ab’)_2_ were determined to be 92.6% (**Figure S1E**) and 93.7%, respectively (**Figure S2F**). These results collectively demonstrated that the purity of IgG and F(ab’)_2_ products were obtained from equines.

### *In vitro* characterization of equine IgG and F(ab’)_2_

**Figure 1:**
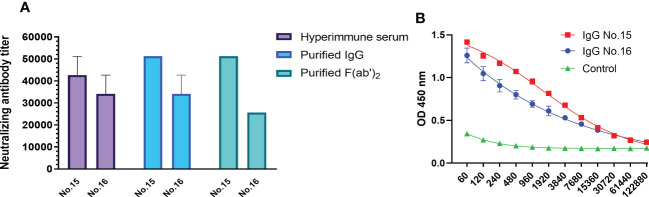
*In vitro* characterization of purified equine immunoglobulin against SARS-CoV-2. **(A)** The neutralizing titers of hyperimmune serum, purified IgG, and F(ab’)_2_ derived from equine No. 15 and No. 16 were tested with wild type SARS-CoV-2 Wuhan 01. The serum neutralizing antibody titer was defined as the reciprocal of the highest dilution showing a 100% CPE reduction compared to the virus control. **(B)** The titers of purified SARS-CoV-2-specific IgG in equine sera were examined *via* RBD-capture ELISA. Two repeated tests were performed on each sample.

Neutralizing activity against SARS-CoV-2 is an indicator of functional antibodies that confer a protective immune response. The binding activity to RBD and neutralizing activity of equine IgG and F(ab’)_2_ were investigated. The RBD protein produced by the prokaryotic expression system was used to detect the binding ability of purified IgG. The results showed that the ELISA titer of IgG was 1:12,280 (**Figure 1B**). Hyperimmune serum, purified IgG and F(ab’)_2_ derived from the No. 15 and No. 16 equines were selected to evaluate whether the purification operation affected the neutralizing ability. As shown in **Figure 1A**, purified IgG and F(ab’)_2_ of equine No. 15 were found to possess similar neutralizing activity levels, with a neutralizing titer of over 50,000, while the titer of equine No. 15 serum products was over 40,000. The neutralizing titer of serum and purified IgG derived from equine No. 16 was over 30,000, but the purified F(ab’)_2_ has a slightly decreased neutralizing titer of over 20,000. No significant differences in neutralizing activity between hyperimmune serum and purified equine immunoglobulins were found after purification. These results indicated that the preparation and purification processes do not adversely affect the neutralizing capacities of these antibodies.

### Broad-spectrum neutralizing activity test

**Figure 2:**
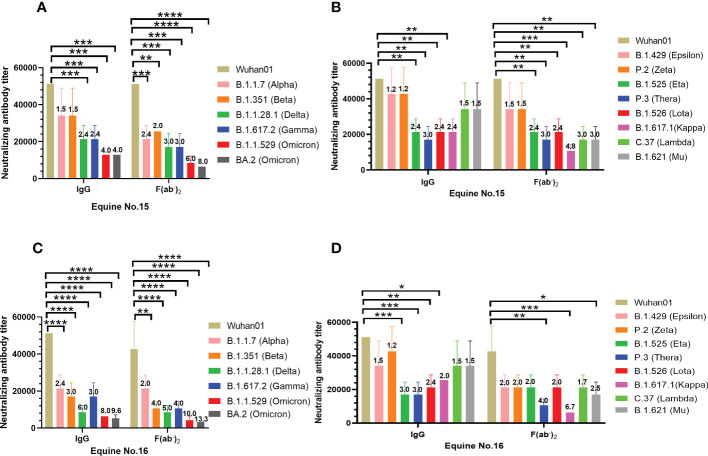
Broad-spectrum neutralizing activity test against SARS-CoV-2 VOC and VOI. The neutralizing antibody titers were calculated as the highest dilution of sera that completely inhibited virus-caused CPE. The serum neutralizing antibody titer was defined as the reciprocal of the highest dilution showing a 100% CPE reduction compared to the virus control. **(A)** Neutralizing antibody titers of purified IgG and F(ab’)_2_ of equine No.15 against SARS-CoV-2 VOC; **(B)** Neutralizing antibody titers of purified IgG and F(ab’)_2_ of equine No.16 against SARS-CoV-2 VOC; **(C)** Neutralizing antibody titers of purified equine immunoglobulin of equine No.15 against SARS-CoV-2 VOI; **(D)** Neutralizing antibody titers of purified equine immunoglobulin of equine No.16 against SARS-CoV-2 VOI. Comparison to the wild type SARS-CoV-2 Wuhan01, the number above the column represented the fold by which the neutralizing titer of the IgG or F(ab’)_2_ was weakened by the SARS-CoV-2 VOC and VOI. Samples were processed in triplicate, and error bars indicate standard error. Data are presented as the mean ± SEM. (*P < 0.05, **P < 0.01, ***P < 0.001, ****P < 0.0001).

**Figure 3:**
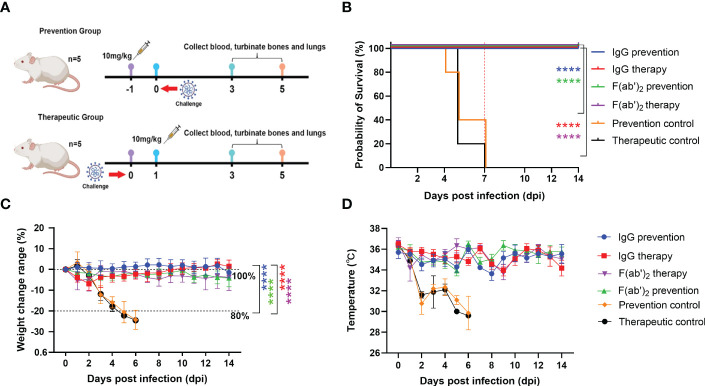
Evaluation of the protective efficacy of purified equine immunoglobulin in a mouse model. Groups of 13 BALB/c mice were administered with IgG or F(ab’)_2_ at 1 day before mouse-adapted SARS-CoV-2 (BMA8) infection or 1 dpi with BMA8. Each mouse was given 250 µg of antibody at a dose of 10 mg/kg. BALB/c mice were challenged intranasally with a lethal dose 50 LD_50_ of BMA8 before treatment or after administration. The survival rate, weight change, body temperature and clinical scores of BALB/c mice were monitored daily after SARS-CoV-2 BMA8 infection. **(A)** Schematic diagram of the administration of equine immunoglobulin drugs and virus challenge procedure; **(B)** Survival rate. **(C)** Percent weight change. **(D)** Body temperature change. Body weight change of mice in a with comparison to isotype control was measured by repeated measurements two-way analysis of variance (ANOVA) with Tukey’s *post hoc* test. Data are mean ± s.e.m. of each experimental group. (****P < 0.0001).

To evaluate the broad-spectrum neutralizing activity of equine immunoglobulin, VSV-vectored pseudoviruses of the five SARS-CoV-2 mutants recommended by the WHO were applied. **Table S1** shows the source of each variant of VOCs and VOIs and the specific information of mutation sites on the S protein. It is not difficult to find that most variants of VOCs and VOIs shared D614G mutation sites since the D614G mutation occurred in the B.1.1.7 variant. Additionally, the N501Y mutation was identified in most SARS-CoV-2 VOCs except for the B.1.617.2 variant. N501Y and D614G mutations were stable in the Omicron variant (BA.1 and BA.2), which has recently continued to circulate around the world (**Table S1**). The results of the neutralization assay suggested that IgG or F(ab’)_2_ derived from equine No. 15 and No. 16 have similar neutralizing activity against SARS-CoV-2 Wuhan01 (**Figures 2A, B**). The mean neutralizing titer of IgG from the two equines was 1:51,200, while the mean neutralizing titers of F(ab’)_2_ were 51,200 and 42,000, respectively. However, the SARS-CoV-2 VOCs defined by the WHO, including Alpha B.1.1,7 (Alpha), B.1.351 (Beta), B.1.617.2 (Delta), P.1 (Gamma), and B.1.1.529 (Omicron), weakened the neutralizing effect of IgG and F(ab’)_2_ to varying degrees. Compared to SARS-CoV-2 Wuhan01, the VOCs weakened the neutralizing titer by 1.5~4.0 folds of IgG and 2.4~8.0 folds of F(ab’)_2_ for No.15 equine (**Figure 2A**), 2.4~9.6 folds of IgG and 2.0~13.3 folds of F(ab’)_2_ for No.16 equine (**Figure 2C**). VOIs weakened the neutralizing titer by 1.2~3.0 folds of IgG and 1.5~4.8 folds of F(ab’)_2_ for No.15 equine (**Figure 2B**), 1.2~3.0 folds of IgG and 1.7~6.7 folds of F(ab’)_2_ for No.16 equine (**Figure 2D**). Among the VOCs, regardless of whether they were IgG or F(ab’)_2_, Omicron had the most significant neutralization weakening ability, with a mean neutralizing titer of 12,800 for No. 15 (**Figure 3A**) and 4,200 for No. 16 (**Figure 3C**). IgG and F(ab’)_2_ products still maintained a strong neutralizing effect. These results indicated that equine polyclonal antibodies against SARS-CoV-2 have potential broad-spectrum neutralizing activity against SARS-CoV-2 variants. For the VOIs, IgG and F(ab’)_2_ of No. 15 equine have a neutralizing capability against pseudoviruses of B1,1,42 (Epsilon) and P.2 (Zeta) similar to that of the parental virus SARS-CoV-2 Wuhan01. Meanwhile, other variants of VOIs to a lesser extent weakened the neutralizing ability (**Figure 2C**). In comparison to equine No. 15, the neutralizing ability of equine No. 16 IgG or F(ab’)_2_ was slightly weaker than that of equine No. 15 (**Figure 2D**). In the following study, we selected the equine serum product of horse No. 15 to evaluate the protective efficacy in rodent models.

### Protection efficacy test of equine immunoglobulin IgG and F(ab’)_2_ in a mouse model

The half-lives of IgG and F(ab’)_2_ were assessed in guinea pigs and found to be 5~6 days and 24~48 h, respectively (**Table 1**). To verify the protective effect of equine antibodies *in vivo*, purified IgG and F(ab’)_2_ were given to wild-type (WT) BALB/c mice by intraperitoneal injection with 250 µg per dose (10 mg/kg) at 1 day post-infection (dpi) or 1 day preinfection with a lethal dose mouse-adapted SARS-CoV-2 (**Figure 3A**). The results indicated that all BALB/c mice injected with IgG or F(ab’)_2_ survived after SARS-CoV-2 infection, while the prevention control and treatment control succumbed to the infection at 4~7 dpi and 5~7 dpi, respectively (**Figure 3B**). Throughout the challenge experiment, all mice in control group had obvious weight loss. At the endpoint of the study, the weight of the surviving mice recovered to their original weight, and the weight of the IgG treatment group increased by 1.50 ± 3.04% (mean ± SD). The body weight of the two control groups dropped to 80% of the initial weight within 2~5 dpi (**Figure 3C**). The mice were euthanized if their body weight was reduced by 20% based on the requirements of the Animal Experimentation Committee. The results of temperature changes showed that the body temperature of mice decreased continuously after SARS-CoV-2 infection, and the temperature changes in the control group were 5 ± 1.55°C in the prevention control group and 5 ± 1.14°C in the therapeutic control group. The body temperature of the prevention group and therapeutic group fluctuated within a normal range. The temperature changes were 0.14 ± 1.09°C in the IgG prevention group and 0.44 ± 0.62°C in the F(ab’)_2_ prevention group; the temperature changes were 2.28 ± 0.82°C in the IgG treatment group and 1.56 ± 0.67°C in the F(ab’)_2_ treatment group (**Figure 3D**).

**Table 1:** Determination of half-life of purified equine IgG and F(ab’)_2_ in guinea pigs.

Group	Guinea Pig Number	Neutralization titer										
		0h	1h	4h	8h	12h	24h	48h	72h	96h	120h	144h
IgG	No.1	–	1:1280	1:1280	1:2560	1:2560	1:2560	1:2560	1:1260	1:640	1:640	1:320
	No.2	–	1:1280	1:1280	1:2560	1:2560	1:2560	1:1280	1:1280	1:640	1:640	1:320
	No.3	–	1:640	1:640	1:1280	1:1280	1:2560	1:1280	1:640	1:640	1:320	1:160
	No.4	–	1:1280	1:1280	1:1280	1:2560	1:1280	1:1280	1:640	1:640	1:320	1:160
	No.5	–	1:1280	1:1280	1:2560	1:1280	1:1280	1:1280	1:1260	1:1260	1:640	1:320
F(ab’)_2_	No.1	–	1:1280	1:1280	1:1280	1:640	1:160	1:80	1:20	–	–	–
	No.2	–	1:1280	1:2560	1:640	1:640	1:160	1:40	1:20	–	–	–
	No.3	–	1:1280	1:1280	1:640	1:160	1:160	1:40	–	–	–	–
	No.4	–	1:1280	1:1280	1:640	1:320	1:160	1:40	–	–	–	–
	No.5	–	1:1280	1:2560	1:1280	1:320	1:80	1:40	–			

### Hematology test

**Figure 4:**
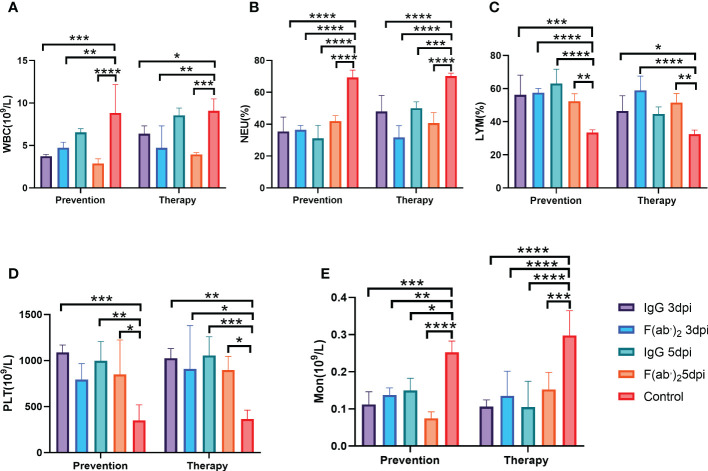
Blood counts in SARS-CoV-2-infected mice. The hematological values of BALB/c mice were analysed, including lymphocyte (LYM), neutrophil percentage (Neu%), monocytes (Mon), platelet count (PLT) and white blood cell count (WBC), at 3 dpi after SARS-CoV-2 BMA8 infection. Four infected mice were sacrificed at 3 dpi to collect the whole blood for blood counts test. **(A)** White blood cell (WBC) count; **(B)** neutrophil (Neu) percentage; **(C)** lymphocyte (LYM) percentage; **(D)** platelet (PLT) **(E)** Monocyte(Mno). Data are presented as the mean ± SEM (n=4). (*P < 0.05, **P < 0.01, ***P < 0.001, ****P < 0.0001).

Hematological analysis revealed that BMA8-infected BALB/c mice in the control group showed a moderate increase in white blood cell (WBC) count (**Figure 4A**), with significant decreases in the percentage of lymphocytes (LYM%) (**Figure 4C**) and monocytes (Mon%) (**Figure 4E**) and a decrease in platelet count (PLT) (**Figure 4D**) at 3~5 dpi in comparison to all treatment groups. A marked increase in the percentage of neutrophils (Neu%) (**Figure 4B**) was also observed in the control group rather than the treatment groups injected with IgG or F(ab’)_2_, which is a typical severe characteristic of SARS-CoV-2 infection observed in clinical COVID-19 patients.

### Viral load

**Figure 5:**
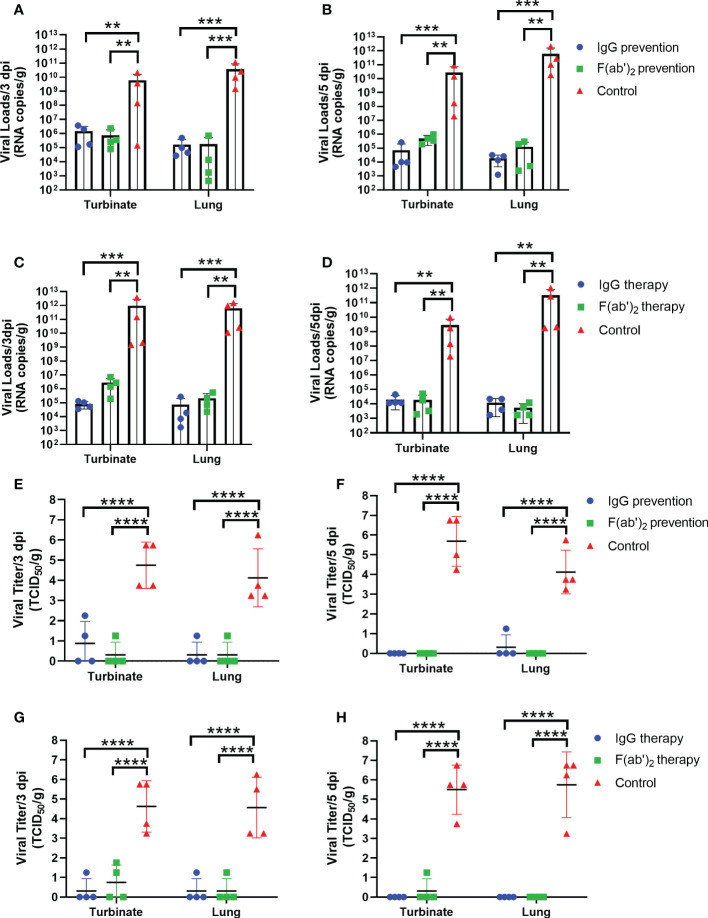
Viral loads in the upper and lower respiratory tracts of mice. Four infected mice were sacrificed at 3 dpi and 5 dpi, respectively, and the turbinate and lungs were harvested to analyze the viral RNA loads by RT‒qPCR and TCID_50_. **(A)** The viral load quantified by RT‒qPCR at 3 dpi in the prevention group. **(B)** The viral load quantified by RT‒qPCR at 5 dpi in the prevention group. **(C)** The viral load quantified by RT‒qPCR at 3 dpi in the treatment group. **(D)** The viral load quantified by RT‒qPCR of the turbinate and lungs at 5 dpi in the treatment group. **(E)** The viral load confirmed by TCID50 at 3 dpi in the prevention group; **(F)** The viral load confirmed by TCID_50_ at 5 dpi in the prevention group; **(G)** The viral load confirmed by TCID_50_ at 3 dpi in the treatment group; **(H)** The viral load confirmed by TCID_50_ at 5 dpi in the treatment group. Data are presented as the mean ± SEM (n=4). (**P < 0.01, ***P < 0.001, ****P < 0.0001).

To assess the bio-distribution of SARS-CoV-2 infection in the upper and lower respiratory tracts, the turbinates and lungs were harvested at selected times (3 dpi and 5 dpi) after infection, and viral RNA (vRNA) was quantified by RT-qPCR and TCID_50_. As shown in **Figure 5**, there were much higher levels of vRNA loads in the lung and turbinate at 3 and 5 dpi in both the prevention control group and the treatment control group than in all administration groups. The vRNA loads reached 10^5^~10^11^ copies/g at 3 dpi (**Figure 5A**) and 10^7^~10^11^ copies/g at 5 dpi (**Figure 5B**) in the prevention control group, while the viral loads reached 10^8^~10^12^ copies/g at 3 dpi (**Figure 5C**) and 10^7^~10^11^ copies/g at 5 dpi (**Figure 5D**) in the treatment control group. Regardless of the prevention group or the treatment group, no significant differences in viral copies between IgG and F(ab’)_2_ were found at 3 dpi or 5 dpi. The TCID_50_ results tended to coincide exactly with the RT-qPCR results, and significantly higher viral titers were confirmed in all control groups (**Figures 5E–H**). Although the treated mice were positive for vRNA in the turbinates and lungs, infectious viruses were not recovered from those tissues from most of the surviving animals at 5 dpi (**Figures 5F, H**). Taken together, the above results illustrated that equine IgG or F(ab’)_2_ treatment significantly inhibited viral replication in the upper and lower tracts, which may block shedding of infectious SARS-CoV-2.

### Histopathology and immunohistochemistry findings in the internal organs of mice

**Figure 6:**
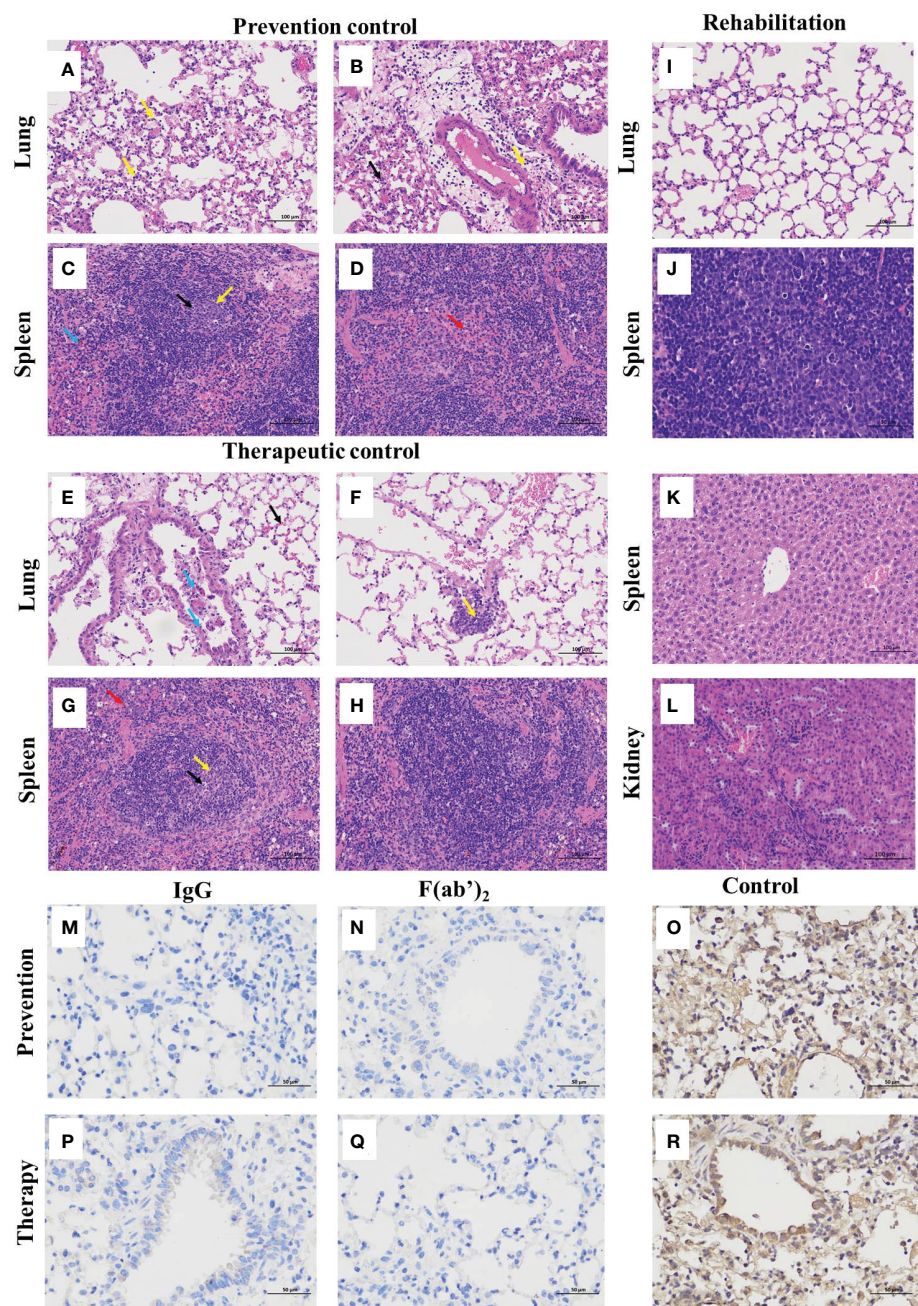
Histopathological and immunohistochemistry findings in SARS-CoV-2-infected mice. The lungs and spleens were collected from the control mice infected with SARS-CoV-2 without equine immunoglobulin drug injection at 3dpi, and the lungs, spleens, livers and kidneys were harvested from recovered mice. After each tissue was embedded in paraffin, the sections were sectioned for HE staining. **(A, B, E, F)** Lung tissue changes of control mice were characterized by more necrotic epithelial cells (blue arrow), a small amount of neutrophil infiltration, and perivascular edema with a small amount of inflammatory cell infiltration in the local alveolar cavity (yellow arrow). **(C, D, G, H)** Spleen tissue changes of control mice were characterized with spotted apoptosis of lymphocytes, nuclear pyknosis and deep staining or fragmentation in the spleen nodules (black arrows), and the expansion of germinal centers (yellow arrow), scattered neutrophils mostly seen in the red pulp granulocyte infiltration (red arrow), and more brown‒yellow particles in the red pulp (blue arrow). **(I-L)** The basically normal structure of the lung, spleen liver, and kidney tissues were found in administration groups given equine IgG or F(ab’)_2_. The figure showed immunohistochemistry (IHC) labeling against SARS-CoV-2 N. **(M)** Viral antigen was not detectable in prevention group given purified IgG; **(N)** Viral antigen was not detectable in prevention group given purified F(ab’)_2_; **(O)** Viral antigen was detected for positive in prevention control group; **(P)** Viral antigen was not detectable in treatment group given purified IgG; **(Q)** Viral antigen was not detectable in treatment group given purified IgG F(ab’)_2_; **(R)** Viral antigen was detected for positive in treatment control group. (scale bar = 100 μm).

The gross pathology and histological assays were evaluated to check the pathological changes in the lungs at 3 dpi. The pathological section of the lung from controlled animals showed that the tissue airway epithelial cells were tightly arranged, and there was no obvious epithelial cell degeneration and necrosis; more necrotic epithelial cells (blue arrows) can be found in individual airway cavities in the local tissues (**Figure 6E**), and the number of airway epithelial cells were reduced; a large range of alveolar cavities were narrowed, and a small amount of alveolar wall capillary congestion (black arrow), and a small amount of neutrophil infiltration was obvious and perivascular edema with a handful of inflammatory cell infiltration (yellow arrow) in the local alveolar cavity (**Figures 6A, B, F**); spleen result prompted that the red pulp and white pulp were clearly demarcated and spotted apoptosis of lymphocytes, nuclear pyknosis and deep staining or fragmentation (black arrows) were shown in the spleen nodules, and the expansion of germinal centers (yellow arrows) could be seen locally (**Figures 6C, G**); scattered neutrals were mostly seen in the red pulp granulocyte infiltration (red arrow) (**Figure 6D**); more brown‒yellow particles (blue arrow) were found in the red pulp (**Figures 6G, H**).

The lungs of the surviving mice showed that the bronchial epithelial structure was intact, the epithelial cells were normal and tightly arranged, and the alveolar structure was clear and not significantly thickened without any obvious inflammation (**Figure 6I**). The splenic tissue capsule was composed of dense connective tissue with uniform thickness and rich in elastic fibers and smooth muscle fibers. The capsule connective tissue extended into the spleen to form trabeculae, with no obvious abnormalities. Hepatic cords were arranged neatly, with no obvious expansion or squeezing of liver sinusoids (**Figure 6J**). The liver lobule was clearly demarcated, and the staining nuclei in the liver cells were blue. The nuclei were large, round, and centered, with little heterochromatin and light staining, and the nucleoli could be clearly seen (**Figure 6K**). The renal tissue was evenly stained, the renal cortex and medulla were clearly demarcated, the glomerulus was normal in shape and structure, and the epithelial cells of renal tubules were closely arranged without obvious inflammation (**Figure 6L**).

To further identify the effectiveness of purified equine immunoglobulin IgG and F(ab’)_2_, after combining the observation of pathological sections, we continued to perform a detailed histopathological assessment of the lungs of the mice at 5 dpi after challenge using immunohistochemistry by detecting SARS-CoV-2 nucleocapsid. Regardless of the prevention group or the treatment group, after the application of IgG or F(ab’)_2_, the mouse lungs were normal and no virus was detectable (**Figures 6M, N, P, Q**). SARS-CoV-2 nucleocapsid was detected in the lung tissue by immunohistochemistry (IHC) staining, especially in conducting airway epithelia and in the alveoli (**Figures 6O, R**). Based on the above histopathological results, equine immunoglobulin IgG and F(ab’)_2_ could effectively clear SARS-CoV-2 in the lungs of mice.

### Protection efficacy test of equine immunoglobulin IgG and F(ab’)_2_ in the golden hamster model

**Figure 7:**
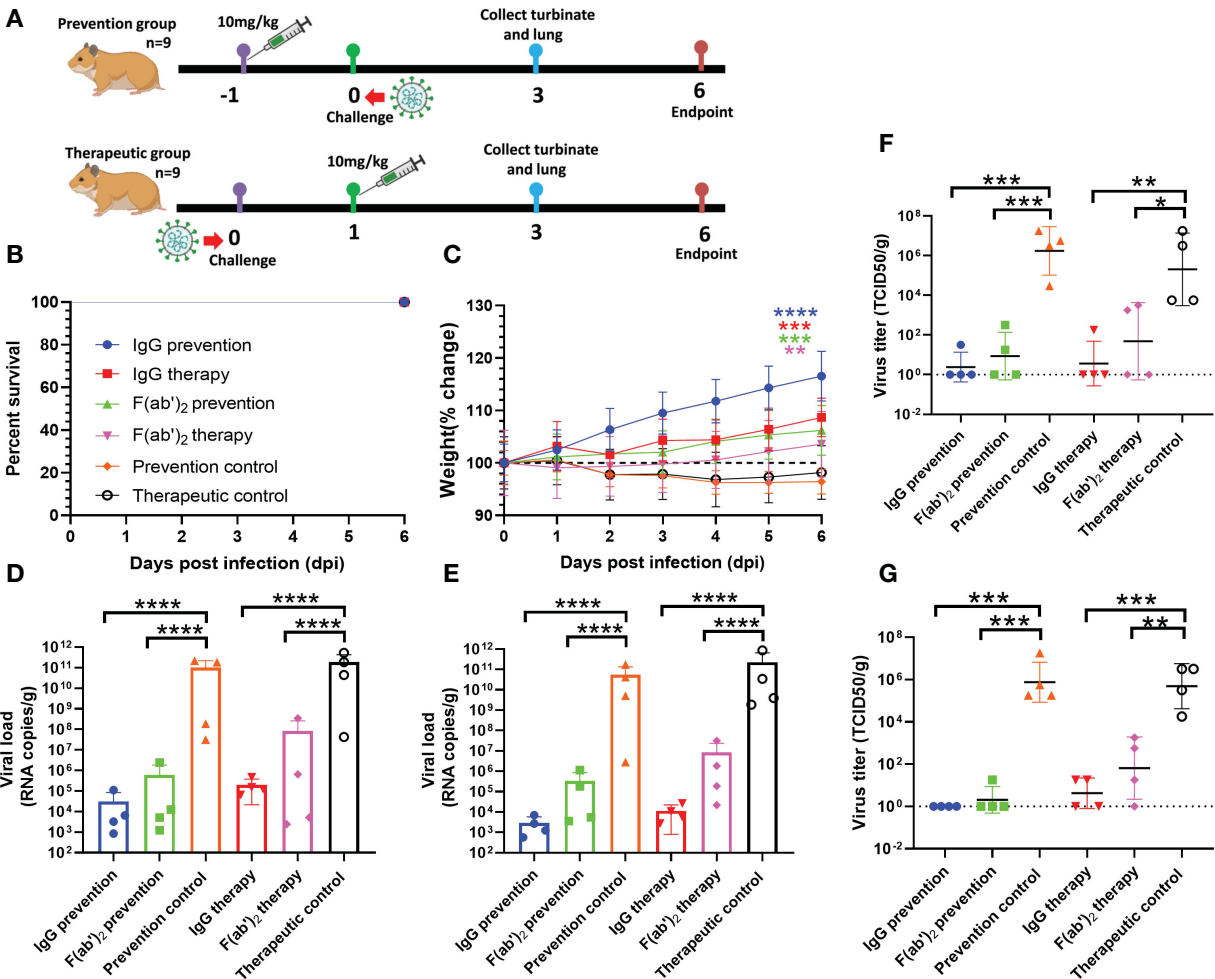
Evaluation of the protective efficacy of purified equine immunoglobulin in the golden hamster model. Each golden hamster was given 500 µg of antibody at a dose of 10 mg/kg. Groups of golden hamsters were infected intranasally with 1,000 TCID_50_ of wild-type SARS-CoV-2 Wuhan 01 before treatment and or after administration. The survival rate and weight change of BALB/c mice were monitored daily after SARS-CoV-2 Wuhan01 infection. Four infected golden hamsters in each group were sacrificed at 3 dpi, and the turbinate and lung samples were collected to analyze the viral RNA loads by RT‒qPCR and TCID_50_, respectively. **(A)** Schematic diagram of the administration of equine immunoglobulin drugs and virus challenge procedure. **(B)** Survival rate. **(C)** Percent weight change; Body weight change of mice in a with comparison to isotype control was measured by repeated measurements two-way analysis of variance (ANOVA) with Tukey’s *post hoc* test. Data are mean ± s.e.m. of each experimental group. **(D)** The viral loads of turbinate were quantified by RT‒qPCR at 3 dpi in each group; **(E)** The viral loads of lung were quantified by RT‒qPCR at 3 dpi in each group; **(F)** The viral loads of turbinate were determined by TCID_50_ at 3 dpi in each group; **(G)** The viral loads of lung were determined by TCID_50_ at 3 dpi in each group. Data are presented as the mean ± SEM (n=5). (*P < 0.05, **P < 0.01, ***P < 0.001, ****P < 0.0001).

The immunocompetent Syrian golden hamster has also been used to evaluate equine pAb activity against SARS-CoV-2 infection in the upper and lower respiratory tracts (40, 41). We used this animal model to independently assess the inhibitory activity of equine IgG or F(ab’)_2_. Weights were followed for 6 days, and then tissues were harvested for virological, histopathology and immunohistochemistry analysis. The treatment strategy is shown in **Figure 7A**. At 500 μg of equine immunoglobulins, hamsters treated with equine immunoglobulin IgG or F(ab’)_2_ and infected with wild-type SARS-CoV-2 Wuhan01 showed a 100% survival rate (**Figure 7B**), protection against weight loss (**Figure 7C**), and reduced viral burden levels in the lungs and nasal swabs compared to the independent control (**Figures 7D, E**). Correspondingly, RT‒qPCR and TCID_50_ analysis showed reduced SARS-CoV-2 RNA replication in the lungs of hamsters treated with equine immunoglobulin IgG or F(ab’)_2_. Although treated animals were positive for vRNA in turbinates and lungs by RT-qPCR (**Figures 7D, E**), infectious viruses were not recovered from most of these animals *via* TCID_50_ analysis (**Figures 7F, G**). Overall, when a 500 μg dose of IgG or F(ab’)_2_ was administered, equine pAbs established a trend toward protection against weight loss in hamsters infected with SARS-CoV-2 Wuhan01.

For the surviving hamster, the structure of the lung tissue was basically normal, and the alveolar wall in the visual field was uniform without thickening. The alveolar outline was clear with normal size. The alveolar epithelial cell nucleus was round with no necrosis; no inflammatory cell infiltration was found in the lung parenchyma (**Figures S3A, E**). For the control group, the lung tissue structure was severely abnormal (**Figures S3B, F**), and proliferation of alveolar epithelial cells is shown by the yellow arrow (**Figure S3B**). A small amount of inflammatory cell infiltration and hemorrhage was found in the lung parenchyma, as shown by the red arrow (**Figure S3F**). Immunohistochemistry results indicated that SARS-CoV-2 was negative in all treatment groups (**Figures S3C, G**) and was detectable in all control groups (**Figures S3D, H**).

## Discussion

The emergence and dissemination of SARS-CoV-2 and the resulting COVID-19 pandemic triggered a global public health crisis. Although several COVID-19 vaccines have been approved for clinical use, demand far exceeds supply, and access to them is inequitable. Moreover, the appearance of SARS-CoV-2 variants and reports of reinfections associated with immune escape highlight the urgent need for effective and broad coverage COVID-19 therapeutics. Of note, the emergence of antibody-resistant SARS-CoV-2 variants that might limit the therapeutic usefulness of monoclonal antibodies can be mitigated by the use of polyclonal antibodies or monoclonal antibody combinations that simultaneously target distinct neutralizing epitopes. A previous study confirmed that the delta VOC has resistance to therapeutic monoclonal antibodies due to binding impairment to the spike protein, indicating increased immune escape (42). Furthermore, innovative therapies such as mAb cocktails of COVID-19 will probably be too expensive for public use in the face of such large numbers of hospitalized infected people in desperate need of drug treatment. Polyclonal antibodies (pAbs) and convalescent plasma are limited to wide application because it is difficult to simultaneously satisfy two strict donor rigorous health conditions with free blood-borne pathogens and high levels of neutralization anti-SARS-CoV-2 antibodies (43). Additionally, the therapeutic strategy of plasma transfusion from convalescent patients cannot meet the large demand for plasma from severe COVID-19 patients. The new generation of processed and purified equine pAbs containing highly purified IgG or F(ab’)_2_ fragments was demonstrated to be safe and well tolerated. Equine pAbs are easy to manufacture, allowing fast development and scaling up for treatment (44).

Equine polyclonal immunoglobulins with Fc cleavage have been approved for the treatment of snake envenomation or exposure to rabies virus (45, 46). Polyclonal equine antibodies consisting of a wider range of antibody types can be induced because intact RBDs are applied as immunogens, including all neutralizing and nonneutralizing epitopes. Moreover, RBD without the NTD domain was selected as an immunogen rather than the complete spike protein or the SARS-CoV-2 whole virus in our study to avoid inducing NTD-specific ADE antibodies and the occurrence of antibody-dependent enhancement (ADE). Additionally, optimized hyperimmunization protocols using repeated doses of SARS-CoV-2 RBD protein in the presence of adjuvants likely contributed to the much higher neutralizing potency in comparison to the mAb. It is also worth mentioning that equine pAbs against the SARS-CoV-2 RBD recognize multiple epitopes, which reduce the risk of viral escape. A previous has indicated that a polyclonal approach of equine anti-SARS-CoV-2 F(ab’)_2_ antibodies that achieved a high level of neutralizing potency against all SARS-CoV-2 variants of concern tested including Omicron BA.1, BA.2, BA.2.12 and BA.4/5 (47). Another study also reported that equine polyclonal antibodies (pAbs) have been assessed in clinical trials in Costa Rica against five globally circulating variants of concern: alpha, beta, epsilon, gamma and delta (48). Although the above studies all evaluated the broad-spectrum neutralizing activity of equine antibodies against SARS-CoV-2 variants *in vitro*, the protective effect of antibodies *in vivo* has not been thoroughly explored. In our study, we not only tested neutralizing activity of all SARS-CoV-2 VOCs and VOIs using VSV vectored pseudoviruses, but also evaluated the protective effect of antibodies *in vivo* using two animal models.

Replication-competent pseudovirus-based vesicular stomatitis virus (VSV) vectors have been used as a useful tool to evaluate the neutralizing activity of various kinds of SARS-CoV-2 antibodies, and the results were similar to those of wild-type SARS-CoV-2 (49, 50). Our neutralizing assay of VOCs based on VSV-vectored pseudovirus also supported the above standpoint, in which the purified equine IgG or F(ab’)_2_ fragment still has high neutralizing potency against all SARS-CoV-2 variants of concern, although to a lesser extent, the neutralizing potency of those equine antibody products was weakened by the variants. Mutants B.1.351 and P.1 have been shown to have potential threats to reduce the efficacy of certain vaccines by weakening the neutralizing capability (51–53). The Omicron variant, as the currently circulating strain and the most divergent variant, has been reported to reduce the sensitivity of SARS-CoV-2 Omicron to antibody neutralization elicited by booster vaccination (54). Notably, in our study, the evaluation results of the neutralizing activity of pseudoviruses against IgG antibodies and F(ab’)_2_ showed that IgG has better neutralizing activity against each mutant, especially for SARS-CoV-2 Omicron. However, plasma derived from vaccinated or convalescent individuals or therapeutic mAbs with neutralizing activity has reduced neutralization of multiple SARS-CoV-2 variants efficiently (55–58). A previous study confirmed that the delta VOC has shown resistance to therapeutic monoclonal antibodies due to binding impairment to the spike protein, indicating increased immune escape (42).

In our *in vitro* study, treatment with purified equine IgG or the F(ab’)_2_ fragment was shown to be 100% effective before challenge and 100% effective postexposure in a BALB/c mouse model. IgG and F(ab’)_2_ have a strong capability to inhibit SARS-CoV-2 replication in the upper and lower respiratory tracts in the BALB/c mouse model and hamster model. Many treated animals were positive for vRNA in turbinate and lung samples; however, infectious virus was not recovered from most animals. Detection of vRNA without recovery of infectious virus particles has also been observed with Ebola virus (EBOV) and Nipah virus (59, 60). Therefore, future studies should be performed to confirm how long vRNA may persist despite a lack of viremia and virus shedding. It is interesting to illustrate whether vRNAs remain in circulation for extended periods of time and are generated from ongoing viral replication. It is worth mentioning that SARS-CoV-2-specific non-neutralizing (nnAbs) antibodies in the purified equine IgG-mediated Fc-dependent antibody mechanism may also contribute to survival and virus clearance. Fc-Fcγ receptors are expressed on the surface of various immune effector cells and helper cells, such as monocytes, macrophages and dendritic cells. Activating FcγRs are expressed in various effector cells, such as NK cells and DC cells, which regulate lymphocyte proliferation and differentiation and promote the secretion of cytokines and other inflammatory factors and other immunological functions (61). The protective mechanisms employed by nnAbs depend on engagement with effector cells expressing Fc-Fcγ receptors and consist of antibody-dependent cellular cytotoxicity (ADCC), complement-dependent cytotoxicity (CDC), and antibody-dependent cell-mediated phagocytosis (ADCP) (62). When human neutralizing antibodies were given after infection, intact mAbs reduced SARS-CoV-2 burden and lung disease in mice and hamsters better than loss-of-function Fc variant mAbs. Fc engagement of neutralizing antibodies mitigates inflammation and improves respiratory mechanics (63). A previous study demonstrated that non-neutralizing antibodies against filoviruses, including EBOV and Marburg virus (MARV), mediated protection by Fc-factor function and enhanced the efficacy of other antibodies (64, 65). ADCC-mediated antibodies (ADCC-Abs) can be induced from monovalent inactivated influenza A (H1N1) virus vaccine, and high titers of ADCC-Abs can contribute to lower virus replication and reduced clinical symptoms (62). A similar phenomenon was also found in which nnAbs antibodies elicited by recombinant rabies virus vectored vaccine of Lassa fever virus (LASV) are critical for protection against Lassa fever *via* the ADCC and ADCP pathways (66). In summary, SARS-CoV-2-specific antibodies could limit infection by directing virion neutralization and/or by targeting infected cells for viral clearance through complement or antibody-mediated cytotoxicity/phagocytosis (67). However, beyond neutralization, whether non-neutralizing antibodies in equine polyclonal antibodies mediate the protective effect of COVID-19 still needs to be further verified, and the broad-spectrum antiviral mechanism of a cocktail of antibody treatment drugs, such as equine polyclonal antibodies, which target multiple epitopes of non-neutralizing antibodies and neutralizing antibodies, also needs to be further explored.

Interestingly, the existence of the Fc fragment of IgG was a “two-edged sword”. On the one hand, we will benefit from the Fcγ receptor pathways in driving antibody-mediated antiviral immunity and exclude the possibility of pathogenic or disease-enhancing effects of Fcγ receptor engagement of anti-SARS-CoV-2 antibodies upon infection (68). On the other hand, equine immunoglobulin is a heterologous protein of the human immune system, and the removal of the Fc fragment also avoids the risk of adverse effects of equine immunoglobulin in the human body, eliminating the major concern of ADE. Our study showed that the removal of the Fc fragment did not impair the neutralizing capabilities and cross-reactivity. The ability of F(ab’)_2_ to neutralize SARS-CoV-2 variants and the curative ratio in animal models was similar to that of purified IgG. Nevertheless, it is still unknown whether the protection mechanism mediated by the Fc fragment will be impaired following the absence of the Fc fragment of equine IgG.

In conclusion, the devastation caused by the COVID-19 outbreak and emerging causative variants highlights the deficiencies in available and affordable treatment options. Our study highlighted that purified equine IgG and F(ab’)_2_ fragments were not only a potential therapeutic countermeasure for COVID-19 but also a broad-coverage therapy for low- and middle-income countries to rapidly establish a large-scale manufacturing capacity to prepare antiviral drugs against SARS-CoV-2 variants.

## Materials and methods

### Animals, viruses and cells

Female BALB/c mice aged 9 months, female hartly guinea pigs aged 6~8 weeks, and female golden hamsters aged 4~6 weeks were purchased from Changchun Yisi Experimental Animal Technology Center. Horses aged 5~6 years were kept in Red Hill Horse Farm and given proper animal welfare. BALB/c mouse-adapted SARS-CoV-2 (BMA8), VSV-vectored pseudoviruses of SARS-CoV-2 (VSV-eGFP-SARS-CoV-2) VOCs and VOIs expressing the reporter gene of enhanced green fluorescent protein (eGFP) were generated and stored in the Biosafety Level 3 Laboratory (BSL-3). Our previous study has reported that BMA8 was obtained by passaging the SARS-CoV-2 Wuhan01 in aged mice for 8 generations, which a mouse-adapted virus cause 100% death in aged BALB/c mice. The mouse model was characterized by high viral loads in the upper and lower respiratory tract, accompanied by lymphopenia, and neutrophilia. Q498H (S), A22D (E) and A36V (E) emerged in structural protein, while five mutations were identified in non-structural protein (69). Wild type SARS-CoV-2 Wuhan01 was gifted from Pro.Chenfeng Qin, an isolation derived from a COVID-19 patient of Wuhan (BetaCov/Wuhan/AMMS01/2020). *Spodoptera frugiperda* Sf9 (ATCC^®^ CRL-1711™) insect ovary cells were grown in Sf-900™ II serum-free medium (Life Technologies, San Diego, CA, USA) at 27°C at 200 rpm in suspension culture for baculovirus infection and protein expression. Vero E6 cells (ATCC^®^ CRL-1587™) were cultured at 37°C in Dulbecco’s modified Eagle medium (DMEM) (DMEM; GIBCO, Grand Island, NY) containing 10% fetal bovine serum (Gibco, ThermoFisher, MA, USA) with 5% CO_2_ for BMA8 propagation and titration.

### Protein expression and purification

The complete genome sequence of SARS-CoV-2 was obtained from GenBank (ID: NC_045512.2), and codons in the RBD (aa319~541) were optimized for protein expression in Sf9 cells (Sangon Biotech, Shanghai, CN). The RBD region gene was cloned into the pFastBac1 vector to generate the recombinant plasmid pFastBac1-RBD. pFastBac1-RBD was transformed into *E. coli* DH10Bac competent cells (Life Technologies, San Diego, CA, USA) to produce recombinant bacmid. According to the manufacturer’s instructions, ExpiFectamine Sf transfection reagent (Gibco, Thermo Fisher, MA, USA) was used to transfect recombinant bacmid into Sf9 cells. The recombinant baculovirus rBV-RBD was harvested 4 days after transfection. To generate recombinant RBD protein, Sf9 cells were infected with rBVs in a volume of 300 mL with a cell density of 1~2×10^7^/mL. At 120 h post-infection, cells and debris were removed from the supernatant by centrifugation at 5,000 rpm for 20 min in a JA-10 rotor (Beckman J-20XP, Fullerton, USA). The cell culture supernatant was harvested and subjected to affinity purification through HisPur™ Ni-NTA Spin Columns (Thermo Fisher, MA, USA) to obtain high-purity recombinant RBD protein.

### Detection of RBD protein by western blot analyses

For western blot analysis, 10 ug high-purity recombinant RBD protein samples separated through SDS-PAGE were transferred onto Polyvinylidene fluoride (PVDF) membranes (Immobilin-p, Millipore, USA). The membranes were then blocked with 5% milk blocking buffer in Tris-buffered saline with Tween 20 (TBST) for 1 hour. A 1,000-fold dilution of the mouse polyclonal antibody (SinoBiological, CN) were used to detect the presence of RBD protein. A 10,000-fold dilution of the HRP-labeled goat anti-mouse IgG was utilized to identify and combine the primary antibody (Abcam, USA). The membranes were visualized using Western Blue stabilized substrate (Promega, USA).

### Indirect immunofluorescence test

Briefly, recombinant baculoviruses-infected Sf9 cells were fixed with 80% cold acetone. Next, the cells were subjected to IFA analysis by using 1,000-fold dilution of mouse monoclonal antibodies against SARS-CoV-2 RBD (SinoBiological, Peking, CN). After incubation with primary antibody, the cells were thoroughly washed with PBS and blocked with PBST. Then, FITC-labeled goat anti-mouse IgG (Millipore, Bedford, USA) was added with 0.3% Evans blue for 1 h as a secondary antibody. The plate was placed under a fluorescence microscope (ZEISS Axio Vert. A1, Germany) to observe and capture the fluorescence.

### Equine immunization

Three 5~6-year-old healthy male horses were fed under standard conditions. After emulsifying 1.5 mg of RBD protein in 1 mL PBS with an equal volume of Freund’s complete adjuvant (Thermo Fisher, USA), horses were vaccinated intramuscularly *via* a subcutaneous multipoint injection route. The subsequent immunization dosage was, respectively given 2.5, 2.5, 3.0, and 4.0 mg of protein in 1 mL PBS mixed with equal volume Freund’s incomplete adjuvant (Thermo Fisher, USA) for each horse *via* subcutaneous multipoint injection route. Each horse was immunized 5 times in total at 14-day intervals (**Figure S2A**). One week after each immunization, and the serum was harvested for further use.

### SARS-CoV-2-specific antibody detection by ELISA

The RBD protein expressed in *E. coli* was coated into a 96-well microtiter plate at a final concentration of 1 μg/ml and incubated overnight at 4°C. Subsequently, the plate were blocked with 2% BSA solution for 2 hours at 37°C, 100 μL of serum samples after serial twofold dilutions were added to each well and incubated at 37°C for 2 h. After the cells were thoroughly washed with PBS containing 0.5% Tween-20, 100 μL of 5,000-fold diluted HRP-labeled goat anti-horse antibody was added to each well, and the plates were incubated at 37°C for 1 h. After three washes, 100 μL TMB (3,3’,3,5’-tetramethyl benzidine) was added to each well and incubated for 15 min. Then, 50 μL 0.5 M H_2_SO_4_ solution was added to the well to stop the reaction in each well. The optical density (OD) was measured at 450 nm. The titer result was defined as the inverse of the maximum dilution of serum.

### Preparation of immunoglobulin and F(ab’)_2_ fragments

A 1/2 volume of saturated ammonium sulfate solution was added to an equal volume of serum diluted with saline. The mixture was gently stirred for 30 min at room temperature, centrifuged at 5,000 rpm for 20 min, and 1/3 volume of saturated ammonium sulfate solution was added. The mixture was incubated at room temperature for 30 min and separated at 5,000 rpm for 20 min. The precipitate was dialyzed in saline overnight at 4°C to remove salt.

The pH of equine antiserum was adjusted to 3.3 with 1 mol/L HCl. The equine antiserum was digested at 30°C for 2.5 h with pepsin activated by NaAc solution (pH=3.3) at a final concentration of 10,000 IU/mL. The digestion reaction was terminated by adding 1 mol/L NaOH to the phase solution to adjust the pH to 7.2. Subsquently, the above solution was purified by protein a column and then by protein g column. The purified protein was confirmed by sodium dodecyl sulfate-polyacrylamide gel electrophoresis (SDS-PAGE). The purity of F(ab’)_2_ was detected by a thin slice scan.

### Purification of SARS-CoV-2 specific equine IgG and F(ab’)_2_

Immunoaffinity chromatography was used to purify RBD-specific IgG and F(ab’)_2_. Sepharose 4B gel was utilized to prepare the RBD immunoaffinity column. The gel was resuspended in the coupling solution in which the RBD protein was dissolved and was placed on an overturning shaker overnight at 4°C. Subsequently, the gel was transferred to an empty purification column. After washing with coupling buffer, the gel was resuspended in 2% sodium borohydride (V/V) and incubated at room temperature for 30 min. After washing again, 0.1 mol/L Tris-HCl (pH=8.0) solution was used to resuspend and block at room temperature for 2 h. After washing, the gel was washed alternately with 0.1 mol/L glycine-HCl (pH=2.7) solution and washing buffer. The residual liquid was removed, and 0.02 mol/L PBS was used for equilibration. Total IgG prepared by the precipitation method or F(ab’)_2_ digested by pepsin was mixed and combined with RBD-4B gel overnight at 4°C. RBD-specific equine immunoglobulin IgG and F(ab’)_2_ were eluted with 0.1 mol/L glycine-HCl (pH=2.7). Residual glycine-HCl in the eluent was terminated with 0.1 mol/L Tris-HCl (pH=9.0) to prevent antibody inactivation. After purification, RBD-specific equine immunoglobulin IgG and F(ab’)_2_ were obtained.

### SDS-PAGE and thin layer chromatography

The purified immunoglobulin and F(ab’)_2_ fragment prepared were analyzed by SDS-PAGE. Samples were mixed with SDS-PAGE loading buffer and boiled for 10 min. The prepared samples were electrophoresed through a 10% precast SDS‒PAGE gel. Finally, the SDS-PAGE gel was stained with Coomassie Brilliant Blue staining solution and incubated at room temperature for 30 min. A Tanon 3,500 automatic gel image analysis system (Bio-Equip, Pecking, CN) was used to capture images after decolorization.

### Wild type SARS-CoV-2 and pseudovirus neutralization assay variants

We measured the cross-neutralizing activity of equine antibodies by wild type SARS-CoV-2 Wuhan01 and pseudovirus-based neutralizing assay. After the inactivated serum samples were diluted from 1:40 with a serial twofold dilution, 100 μL of diluted serum samples were added to a 96-well plate, and 100 TCID_50_ wild type SARS-CoV-2 or VSV-eGFP-SARS-CoV-2 was added to each well. 1×10^4^ Vero E6 cells were added to each well after the virus-serum mixture was incubated at 37°C for 1 hour. Cytopathic effect (CPE) was detected under a microscope after the cells were cultured at 37°C for 48~72 hours. The neutralizing antibody titers were defined as the inverse of the highest dilution, which could completely inhibit the production of the cytopathic effect (CPE) compared with viral controls. Virus control and cell control were included in each test.

### Half-life test in guinea pigs

Two groups of 5 inbreed hartly guinea pigs were administered an intraperitoneal (IP) injection of l mL purified equine antisera (neutralization titer 1:20,480) or F(ab’)_2_ (neutralization titer 1:20,480). Blood samples were collected at 0, 1, 4, 8, 12, 24, 48, 72, 96, 120, and 144 h post-injection, and sera were used for determination of neutralization titers against wild-type SARS-CoV-2 Wuhan01. The half-life was defined as the time range post-infection in which during which the serum neutralizing antibody titer decreased by 50%.

### Mouse challenge study

Mice were randomized into six groups of 13. Each mouse was given 250 µg of antibody at a dose of 10 mg/kg. In the prevention group, BALB/c mice were first intraperitoneally given 250 µg purified equine IgG or F(ab’)_2_ and challenged by intranasal administration of 100 TCID_50_ mouse-adapted SARS-CoV-2 in 50 µL DMEM after 24 h. In the treatment group, BALB/c mice were first challenged with 100 TCID_50_ mouse-adapted SARS-CoV-2 in 50 µL DMEM and given 250 µg purified equine IgG or F(ab’)_2_ *via* IP injection 24 h post-infection. At 3 dpi and 5 dpi, four mice in each group were sacrificed. Lungs and turbinates were harvested to test the viral RNA loads, histopathology and immunohistochemistry test. The challenge strain of mouse-adapted SARS-CoV-2 named BMA8 was previously described (69). All mice were monitored for clinical signs of disease, survival rate, weight change and temperature changes.

### Viral load determination by RT-qPCR and TCID_50_

Briefly, viral RNA in each tissue homogenates sample was extracted with an RNeasy Mini kit (QIAGEN, USA) following the manufacturer’s protocol. The viral RNA quantification was performed by RT-qPCR targeting the N gene of SARS-CoV-2. RT-qPCR was performed using One Step PrimeScript RT-PCR Kit (Takara, Dalian, China) with the following primers and probes: NF (5’-GGGGAACTTCTCCTGCTAGAAT-3’); NR (5’-CAGACATTTTGCTCTCAAGCTG-3’); and NP (5’-FAM-TTGCTGCTGCTTGACAGATT-TAMRA-3’). The details can be referred to our previous study (69).

The supernatant of tissue homogenate was serially diluted at a tenfold ratio, and 100μL were added into 96-well cell culture plates that had been plated with Vero E6 cells. The plates were incubated at 37°C for 1 h and then washed 3 times with sterile PBS. 100μL of incomplete medium containing 2% FBS and 1% penicillin‒streptomycin was added to each well of 96-well plate. The median tissue culture infective dose (TCID_50_) was calculated by observing the number of wells with cytopathic effect (CPE) based on the Reed and Muench method after incubating for 72 h.

### Hematological tests

To determine the complete blood cell counts in mice, Blood was collected through the orbital venous plexus, blood was collected through the orbital venous plexus into anticoagulant tubes containing EDTAK2. Blood cell count was carried out under the automatic blood analyzer (BC-5000vet, Mindray, China), and the operation procedures were carried out according to the instrument operation instructions.

### Histopathology and immunohistochemistry

Mice were euthanized at the appointed time. Lungs, spleens, kidneys and livers were collected. Tissue samples from mice were fixed in 10% buffered formalin and embedded in paraffin wax. Lung tissue was stained with hematoxylin and eosin (H&E) for histopathological assessment. For immunohistochemistry (IHC), quenching of paraffin-embedded lung tissues was performed with 3% hydrogen peroxide in methanol for 10 min. The tissue was added with 500-fold dilution of mouse monoclonal anti-SARS-CoV-2 N antibody at 4°C for overnight. After incubation with primary antibody, sections were washed with PBS for 3 times and species-matched secondary antibodies were applied for 2 h at 4°C. The sections were observed under ZEISS Axio Vert. A1 microscope. The details of the histopathology and immunohistochemistry assays can be found in our previous study (69).

### Hamster challenge study

Hamsters were randomized into six groups of 9. Each Syrian hamster was given 250 µg of antibody at a dose of 10 mg/kg. In the prevention group, hamsters were administered purified equine IgG or F(ab’)_2_ by IP injection and challenged with 1,000 TCID_50_ wild-type SARS-CoV-2 Wuhan01 by the intranasal route in a final volume of 100 μL after 24 h. In the treatment group, hamsters were first challenged with 1,000 TCID_50_ SARS-CoV-2 Wuhan01 and given purified equine IgG or F(ab’)_2_ by IP injection 24 hours post-infection. All hamsters were monitored for body weight loss. At 3 dpi, four hamsters in each group were euthanized. Lungs and turbinates were harvested to detect the viral loads, histopathology and immunohistochemistry test.

### Ethical statement

All procedures involving cells and animals were conducted in a biosafety level 3 laboratory (BSL-3) and approved by the animal experimental committee of the Laboratory Animal Center, Changchun Veterinary Research Institute, Chinese Academy of Agricultural Sciences (approval number: IACUC of AMMS-11-2020-020). Animals are given enough drinking water, adequate food and comfortable living conditions in accordance with animal welfare principles.

### Statistical analysis

Figures were generated using GraphPad Prism 8.0 software. Differences between means were evaluated using one-way ANOVA or two-way ANOVA and were deemed significant at P values of 0.05 or less.

## Data availability statement

The original contributions presented in the study are included in the article/**Supplementary Material**. Further inquiries can be directed to the corresponding authors.

## Ethics statement

The animal study was reviewed and approved by Changchun Veterinary Research Institute, Chinese Academy of Agricultural Sciences.

## Author contributions

Conceptualization, YoZ, BL and XX. Methodology, FY. Software, SWe, YiZ. Formal analysis, SWa, QH, BL. Investigation, NF, TW, JL. Resources, JH. Data curation, FY, JB, QH and EL. Writing original draft preparation, FY, JB and EL, SWa. Writing review and editing, FY. Supervision, YoZ, XX and SY. Project administration, JW, XX. Funding acquisition, YoZ. All authors contributed to the article and approved the submitted version.
