# Supplementary material for: Therapeutic equine hyperimmune antibodies with high and broad-spectrum neutralizing activity protect rodents against SARS-CoV-2 infection
Source: Front Immunol. 2023 Feb 17;14:1066730. doi: 10.3389/fimmu.2023.1066730 (PMC9981790; doi:10.3389/fimmu.2023.1066730)
Supplement: Supplementary file 1 [file DataSheet_1.docx]

**Supplementary information**

**Supplementary Table S1. SARS-CoV-2 variants-related information and mutations in spike protein**

| WHO label | Genebank ID | Variant of concern（VOC） | Provenance | Amino acid changes in spike protein |
| --- | --- | --- | --- | --- |
| Wuhan01  Alpha | MZ314997.1  OM616632.1 | --  B.1.1.7 | China  United Kingdom | --  69-70^-^; 144-145^-^; N501Y; A570D; D614G; P681H; T716I; S982A; D1118H |
| Beta | MZ433432.1 | B.1.351 | South Africa | D80A; D215G; 241-243^-^; K417N; E484K; N501Y; D614G;A701V |
| Gamma | MZ477759.1 | P.1/ B.1.1.28.1 | Brazil | L18F; T20N; P26S; D138Y; R190S; K417T; E484K; N501Y; D614G; H655Y; T1027I; V1176F |
| Delta  Omicron | MZ377116.1  OM570278.1 | B.1.617.2  BA.1/B.1.1.529 | India  South Africa | T19R; 157-158^-^; L452R; T478K; D614G; P681R; D950N  A67V; 69^-^; 70^-^; T95I; 142^-^; 143^-^; 144^-^; Y145D; 211^-^; L212I; G339D; S371L; S373P; S375F; K417N; N440K; G446S; S477N; T478K; E484A; Q493R; G496S; Q498R; N501Y; Y505H; T547K; D614G; H655Y; N679K; P681H; N764K; D796Y; N856K; Q954H; N969K; L981F |
| Omicron | OM617939.1 | BA.2 | South Africa | T19I; 24^-^; 25^-^; 26^-^; A27S; G142D; V213G; G339D; S371F; S373P; S375F; T376A; D405N; R408S; N440K; S477N; T478K; E484A; Q493R; Q498R; N501Y; Y505H; D614G; H655Y; N679K; P681H; N764K; D796Y; Q954H; N969K |
| WHO label | Genebank ID | Variant of inierest（VOI） | Provenance | Amino acid changes in spike protein |
| Zeta | MW988205.1 | P.2 | Brazil | L18F; T20N; P26S; F157L; E484K; D614G; S929I; V1176F |
| Epsilon | OM488024.1 | B.1.429 | California | S13I; W152C; L452R; D614G |
| Mu | MW450666.1 | B.1.621 | Columbia | T95I; Y144S; Y145N; R346K; E484K; N501Y; D614G; P681H; D950N |
| Eta | MZ362439.1 | B.1.525 | United States | 69-70^-^; 144^-^; Q52R; E484K; Q677H; D614G; F888L |
| Iota | MZ702241.1 | B.1.526 | United States | T95I; D253G; L5F; S477N; E484K; D614G; A701V |
| Theta | MW896444.1 | P.3/B.1.1.28.3 | Philippines | 141-143^-^; E484K; N501Y; P681H |
| Kappa | MZ571142.1 | B.1.617.1 | India | T95I; G142D; E154K; L452R; E484Q; D614G; P681R; Q1071H. |
| Lambda | MZ496613.1 | C.37 | Peru | G75V; T76I; 246-252^-^; L452Q; F490S; D614G; T859N |

“-” means amino acid deletion

**Supplementary Figures and legends**

**
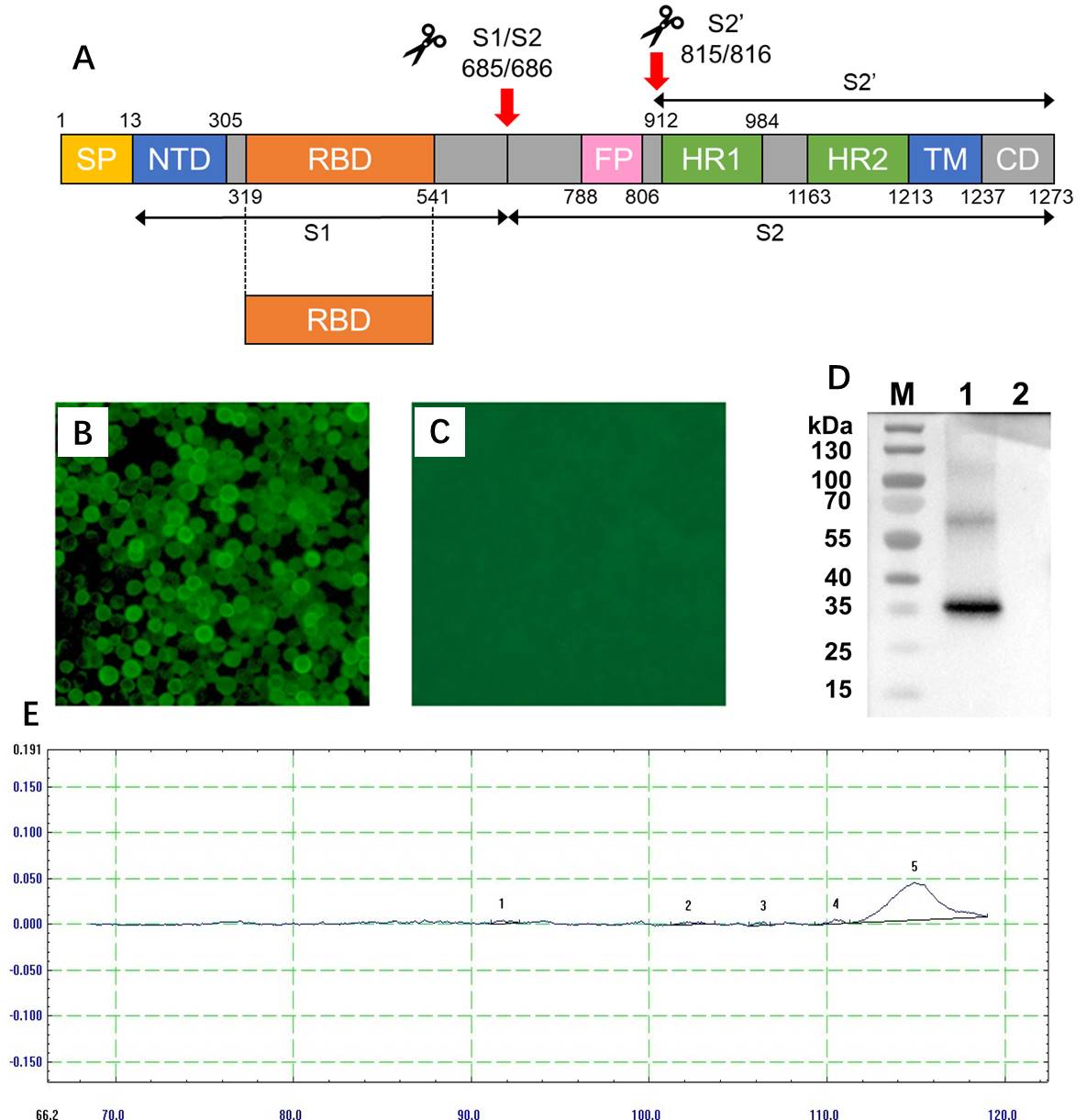
**

**Supplementary Fig.S1 Recombinant RBD protein expression and purification.** (A) The SARS-CoV-2 S protein contains a single peptide; a receptor-binding subunit, S1; and a fusion subunit, S2. The RBD was predicated to include amino acids 319-541 of the S protein.; (B) The Sf9 cells infected recombinant baculovirus expressing protein RBD was confirmed by indirect immunofluorescence (IF); (C) The Sf9 cells infected wild type baculovirus was as a control in IF assay; (D) Recombinant RBD protein was confirmed by western blot assay; (E) The protein purity of the recombinant protein RBD was determined by thin layer chromatogram scanning.

**
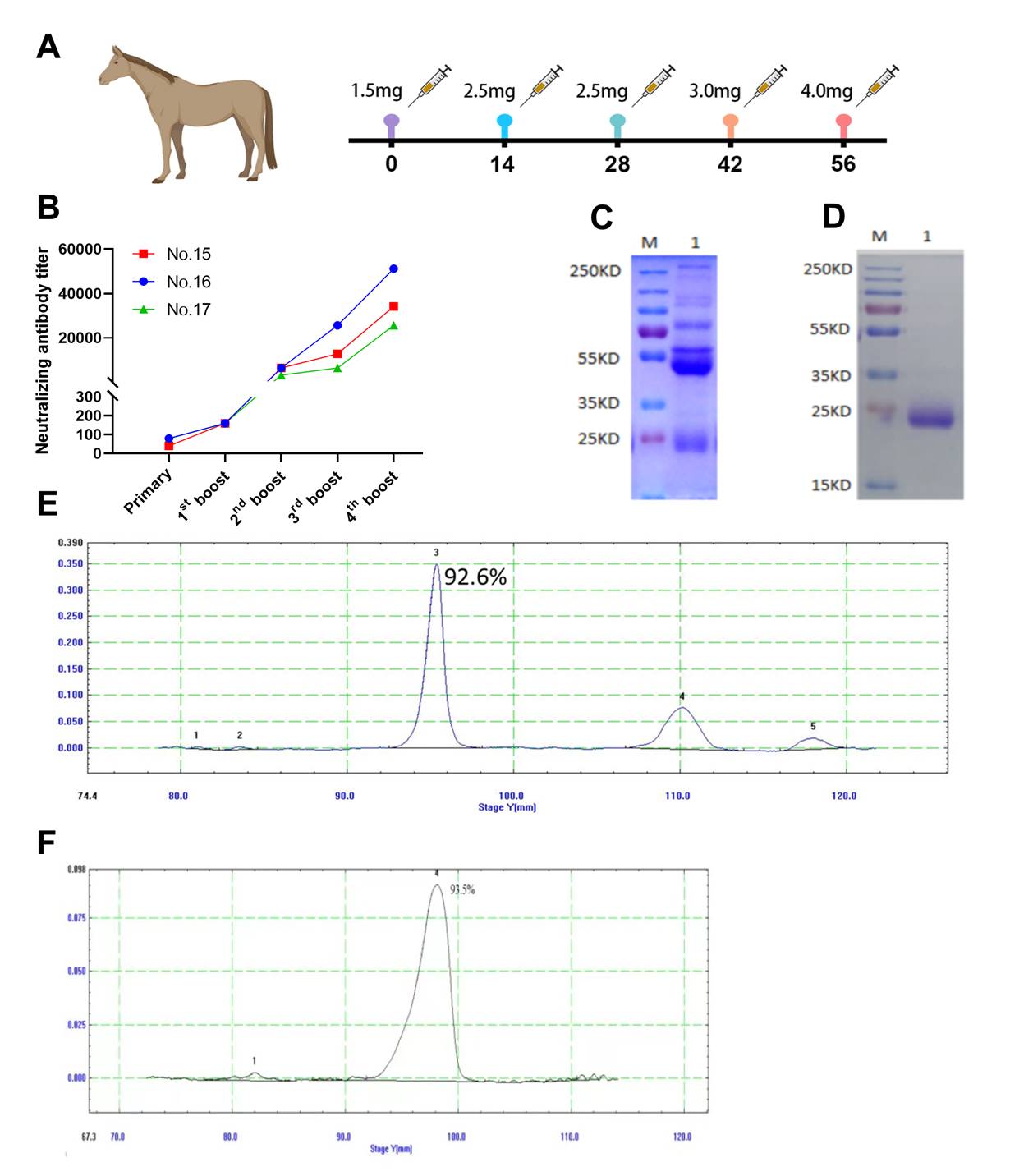
**

**Supplementary Fig.S2** **Equine immunization strategy and characterization of antisera against SARS-CoV-2.** (A) Scheme of equine immunization. Each horse was vaccinated for 5 times, immunized with 1.5 mg, 2.5 mg, 2.5 mg, 3.0 mg, 4.0 mg RBD protein respectively via a subcutaneous multipoint injection routte. The immunization schedule and sampling were shown in the scheme, and the detailed immunization process was described in the Methods section; (B-C) The neutralizing titer of sera from RBD-immunized equines. The serum dilutions ranged from 1:20 to 1:51200. The serum neutralizing antibody titer was defined as the reciprocal of the highest dilution showing a 100% CPE reduction compared to the virus control. _2_; (D) SDS-PAGE and Coomassie blue staining of the purified IgG and F(ab’); (E) The purity of IgG, the purity was determined to be 92.6%; (F) The purity of F(ab’)_2,_ the purity was determined to be 93.5%.

**
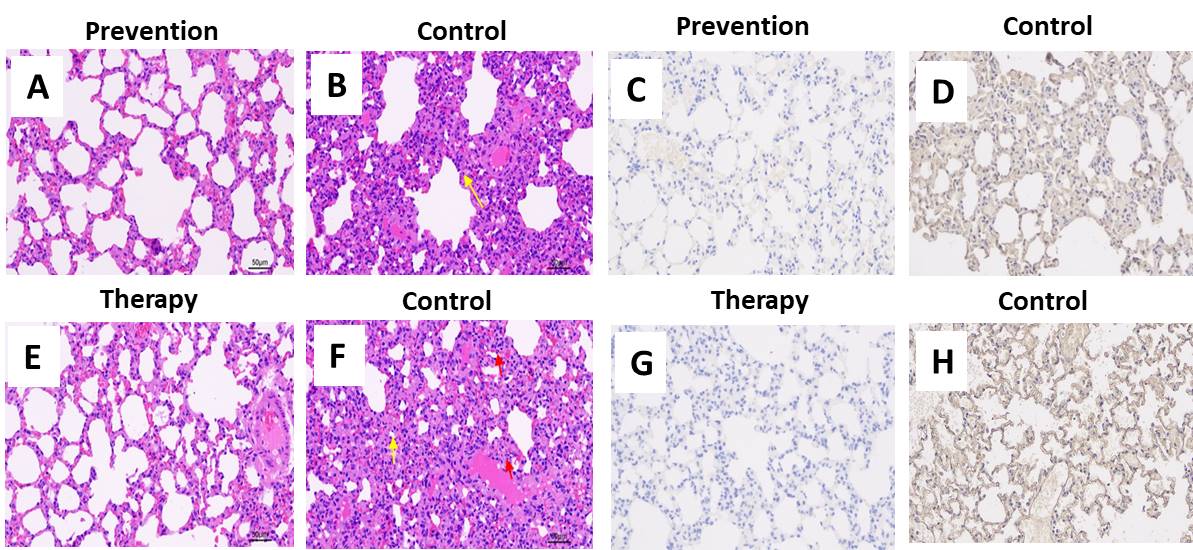
**

**Supplementary Fig.3 Histopathological and immunohistochemistry findings in SARS-CoV-2 infected golden hamster at 3dpi.** (A, E) The basically normal structure of the lung tissue was found in prevention and treatment groups given purified IgG or F(ab’)_2_; (B, F) A small amount of inflammatory cell infiltration and hemorrhage (red arrow), severe abnormality of lung tissue structure, and alveolar epithelial hyperplasia were respectively confirmed in in prevention and treatment control group; (C, G) Viral antigen was not detectable in the lung section of prevention and treatment group; (D, H) Viral antigen was detected for positive in lung section both in prevention control and treatment control group. The figure showed immunohistochemistry (IHC) labeling against SARS-CoV-2 N, Scale bar= 50μm.
